# Short-term direct reciprocity of prosocial behaviors in Japanese preschool children

**DOI:** 10.1371/journal.pone.0264693

**Published:** 2022-03-02

**Authors:** Mayuko Kato-Shimizu, Kenji Onishi, Tadahiro Kanazawa, Toshihiko Hinobayashi

**Affiliations:** 1 Department of Education, Osaka Seikei University, Osaka, Japan; 2 Department of Early Childhood Education, Nara University of Education, Nara, Japan; 3 Graduate School of Human Sciences, Osaka University, Osaka, Japan; 4 Faculty of Health Sciences, Aino University, Osaka, Japan; University of Iowa, UNITED STATES

## Abstract

Direct reciprocity plays an essential role in forming cooperative relationships. Direct reciprocity requires individuals to keep track of past interactions and condition their behavior on the previous behavior of their partners. In controlled experimental situations, it is known that children establish direct reciprocity according to the partner’s behavior, but this has not been verified in real life. This study aims to identify the establishment of short-term direct reciprocity in response to peers’ behaviors among Japanese preschoolers aged 5 and 6. It employs naturalistic observation at a nursery school. In addition, the psychological process for direct reciprocity was examined. The findings demonstrated that after receiving prosocial behavior, the recipient child returned the prosocial behavior more frequently within 7 minutes, compared with control situations; this suggests that 5-to 6-year-olds formed direct reciprocity in the short term when interacting with their peers. Additionally, recipient children tended to display affiliative behavior after receiving prosocial behavior. Positive emotions toward initiating children may have been caused by receiving prosocial behavior, and this psychological change modified short-term direct reciprocity.

## Introduction

Cooperative relations between relatives have been confirmed in many animal species. However, people also form cooperative relations between nonrelatives. One mechanism that is believed to make these cooperative relationships possible is direct reciprocity, selecting people who have supported you in the past and supporting them in return [1, 2]. For direct reciprocity to be established, the exchange of prosocial behaviors between two individuals—returning prosocial behavior to a person who has behaved prosocially towards you—plays an important role. In research targeting adults, direct reciprocity has been demonstrated to function within a cooperative relationship both in experimental settings using computer simulations [1] and real-life [3]. Studying cooperative relations based on direct reciprocity during early childhood will likely make it possible to understand how the capacity to engage in cooperative relationships develops. Several studies have revealed the characteristics of direct reciprocity that young children established.

Strayer [4] made behavioral observations, targeting groups of preschoolers, and reported that the number of prosocial behaviors received between two individuals correlated positively to the number of prosocial behaviors offered by one child to another. In Strayer’s research, there were no controls for the frequency of usual affiliative interactions, so no conclusions could be drawn about whether the direct reciprocity seen in prosocial behaviors was established independently of the young children’s existing familiarity. In contrast, through naturalistic observations in a group childcare setting, Fujisawa et al. [5] demonstrated that direct reciprocity of prosocial behaviors is established in exchanges between 3- to 4-year-olds, independent of usual affiliative interactions. These studies are meaningful as they demonstrate how young children exchange prosocial behaviors in a direct reciprocal manner in the course of exchanges in a natural setting. They also demonstrate that the number of prosocial behaviors received between a pair of young children within a long-term research period correlates with the number of prosocial behaviors offered. However, they do not demonstrate how a young child, the recipient of specific prosocial behavior, selectively returns (or does not return) prosocial behaviors according to the other child’s behaviors.

Several experimental studies have investigated the development of direct reciprocal exchanges that correspond to the other person’s behaviors. Levitt et al. [6] had a pair of 2.5- to 3-year-olds enter two adjoining rooms, handed out a toy to just one child in the pair, and had the mother of the child prompt her child to hand the toy to the other child. Afterward, the researchers handed a toy only to the child who had not been given a toy initially and examined what sort of exchanges could be seen. The results confirmed that, of the ten pairs of young children where one had previously handed out a toy to the other, nine pairs shared the toy in subsequent sessions. This result suggests that direct reciprocal prosocial exchanges are established from the early stages of childhood. However, these young children may have merely behaved reciprocally due to being prompted by their mothers, so we cannot conclude if they had spontaneously acted direct reciprocally or not.

Birch and Billman [7] conducted a trial targeting pairs of 3- to 5-year-olds, in which pieces of candy were handed out unevenly (such as one child getting ten pieces of candy and the other child getting just one), and investigated if the child given lots of candy would give candy to the other child who was not given much. The results demonstrated that if a child had not been given much candy in the previous trial and had received candy from another child, thirteen out of fourteen children would share their candy with the other child in a subsequent trial. In contrast, if a child had not been given much candy in the previous trial and had not received any candy from the other child, only seven out of thirteen children gave candy to the other child in the subsequent trial. This research suggests that, even under circumstances where there is no prompting from adults, young children spontaneously return prosocial behaviors in reciprocally if they are the recipient of prosocial behaviors from the other child. This study, however, adopted an experimental design in which the makeup of the pair changed with each trial, resulting in the drawback that the content of exchanges between the same pairs could not be compared.

Unlike these two studies, House et al. [8] involved children aged 3–7.5 years old as subjects to investigate, in the light of their developmental changes, how the same pair of children exchanged prosocial behaviors in direct reciprocally, without any prompting from adults. Using a card game, the children were asked to choose between a selfish distribution (1 for self, 0 for other) or an equal distribution (1 for self, 1 for other). The results revealed that children aged 5 to 7 were more likely to choose equal option than unequal option, and were more likely to choose equal option if their partner had previously made the same choice. On the other hand, there was no difference between equal and unequal choice for children aged 3 and 4. It indicated that direct reciprocity was more likely to be established with increasing age and that, by the time children reach about 5.5 years old, they can establish stable, direct reciprocity, such as returning (or not returning) prosocial behaviors in response to the other child’s earlier actions and behaviors.

The results of these experimental studies suggest that direct reciprocity that corresponds to the other child’s behaviors already begins to be established in early childhood. However, these studies investigated exchanges between young children in controlled experimental settings, so there are concerns that these exchanges might be unrealistic in natural settings. Moreover, no studies have investigated how young children establish direct reciprocity in response to the peer’s behaviors within their natural exchanges.

To study if in real life young children establishes direct reciprocity in their exchanges with their peers of the same age, it is important to compare the recipient child’s subsequent behaviors between two different sets of circumstances: when a child has demonstrated prosocial behaviors, and when a child has not demonstrated prosocial behaviors. This is because, even if a young child has been the recipient of prosocial behavior from a partner child after they have demonstrated prosocial behavior, we cannot conclude whether this was in return for a specific child’s prosocial behavior unless we compare this with exchanges between the two children in sessions where a young child did not demonstrate prosocial behaviors (a “control setting”). Only by comparing a session after a young child has demonstrated prosocial behavior with a session where a young child has not demonstrated such behavior can we conclude that the other child is likely to return prosocial behavior to a child after the latter has demonstrated prosocial behavior. Therefore, in this study, we created a controlled session and investigated whether direct reciprocity had been established.

This observation method has been applied to studies of the reconciliation behaviors of young children [9–11], and behavioral observational studies of verbal responses from adults made after a child’s finger-pointing [12]. Using this observation method with the control procedure makes it possible to draw a direct comparison between ordinary settings and settings after one child has demonstrated prosocial behavior; it is also possible to examine what sort of influence a child’s prosocial behavior has on the subsequent reaction of the other child with whom exchanges take place.

When using the control procedure, observation of control sessions generally begins with a circumstance in which the child who performs the target behaviors is near a child who is the recipient. This is because in settings immediately after a specific behavior has occurred, the initiating child and the recipient are always in close proximity, and so, unless similar-distance relationships are also established in control sessions, we would not have appropriate control sessions. By establishing several appropriate limitations as the observational conditions of control sessions in this way, the data of a session after a specific behavior and the data of a controlled session become the corresponding data that handle similar child pairs, making it possible to conduct comparisons as the Within-Subject Factor. A variety of individual differences, such as a child’s sociability [13] and self-assertiveness [14], influence prosocial behaviors that occur among peers. With a comparison that uses a pair consisting of a child and their exchange partner as the Within-Subject Factor, it is possible to compare the content of exchanges made between two sessions observed in the same pair; it also becomes possible to consider the influence of individual differences.

There is a possibility that the following factors influence the establishment of direct reciprocity that will be investigated in this study. The first factor is familiarity between a pair. Throughout the preschool period, mutual interactions between young children are influenced by their degree of familiarity in both quality and quantity [15]. Prosocial mutual interactions differ according to the degree of familiarity [16], and several studies have verified that familiarity influences the exchange of prosocial behaviors [5, 7, 17]. The second factor is the difference of usual frequency of receiving prosocial and affiliative behaviors. An observation study conducted at a nursery school demonstrates individual differences in usual frequency of receiving social behaviors such as prosocial and affiliative behaviors [18–20]. In examining prosocial exchanges between children, it is necessary to consider the influence of the factors mentioned above, such as familiarity and usual frequency of receiving social behaviors. In this study, to control for the influence of these factors, we calculated, for each child, the degree of familiarity and usual frequency of receiving social behaviors and included them in our analysis.

In considering behavioral tendencies of prosocial exchanges, it was necessary to consider modeling, a phenomenon whereby changes occur in an observer’s behavior as a result of observing other people’s behaviors as a model. Previous studies have reported that modeling occurs in a child by observing another child or children act in a prosocial manner [21–23]. In other words, a model, or prosocial child, is likely to evoke prosocial behaviors in other children. For direct reciprocity to be established, a child who is the recipient of a prosocial behavior must select a person who has demonstrated the former prosocial behavior and return such behavior. If modeling occurs by observing other prosocial children, and if a child demonstrates prosocial and affiliative behaviors randomly, without selecting a recipient, we cannot say that direct reciprocity has been established that corresponds to the initiating child’s prosocial behavior. In the observation settings in our study, if a child (X) receives prosocial behavior from another child (Y) and attempts to return such behavior but before doing so has proactive behaviors demonstrated by another nearby child (Z), and even if the recipient child (X) had engaged in prosocial behavior afterward to the initiating child (Y), we could not deny the possibility that modeling has occurred due to the behavior of child (Z), so we cannot conclude that the child (X) has selected the other child (Y) who had performed prosocial behavior, and returned such behavior. This was why, in our study, in addition to analyses using all the data, we employed data sets that excluded sessions in which modeling had likely occurred due to the prosocial behaviors of other children nearby to consider the possibility that modeling had occurred.

This study’s objectives were to identify how preschoolers aged 5 and 6 in group childcare settings responded immediately after having prosocial behaviors demonstrated by their peers and investigate if direct reciprocity had been established in the short term. Previous studies have reported that prosocial behaviors that occur between children increase significantly during the preschool period (ages 3–6) [24]. In our study, because it was necessary to observe even greater numbers of prosocial behaviors to perform a quantitative analysis, the subjects included 5- and 6-year-olds who were believed to already demonstrate increased prosocial behaviors. We made observations of sessions after a child demonstrated prosocial behavior to another child, as well as the control sessions, and compared the content of behaviors demonstrated by the recipient child.

**Fig 1:**
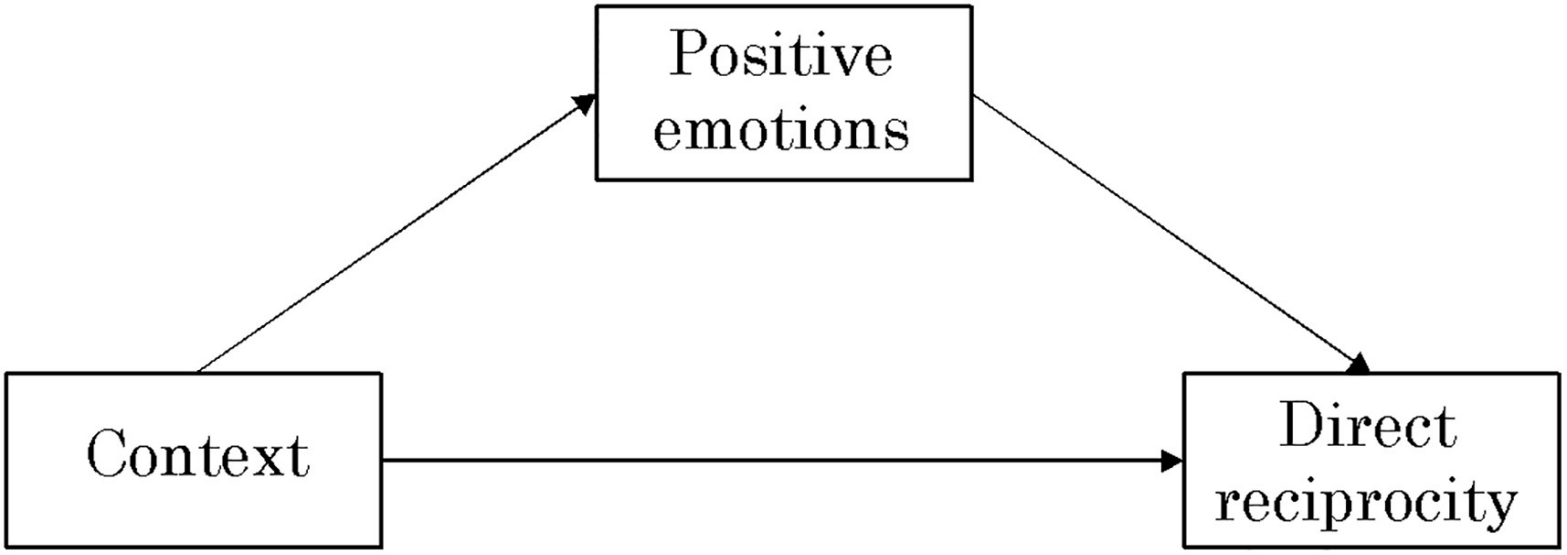
Hypothesized model.

Examining the psychological process for direct reciprocity to be established is important. Children aged 4–5 reportedly explain the reason for performing prosocial behaviors as holding positive emotions towards the other child [25]. Positive emotions, therefore, appear to be one of the psychological processes that promote the occurrence of direct reciprocal exchange. However, as we used naturalistic observations in this study, we could not directly examine a child’s psychological processes. Therefore, we decided to record behaviors to analyze a child’s psychological processes and recorded affiliative behaviors in addition to prosocial behaviors seen in both sessions. Young children hold positive emotions towards others who direct affiliative behaviors toward them [26]. Therefore, affiliative behaviors can be seen as an expression of positive emotions by the recipient child to the initiating child. If compared to the control sessions, a recipient child is more likely to demonstrate affiliative behaviors to the initiating child in sessions after the latter has demonstrated prosocial behavior, we can conclude that positive emotions have been evoked towards the initiating child as a result of being the recipient of prosocial behavior. In consideration of the above, we set up two hypotheses in this study:

(a) If direct reciprocity were established in the short term, prosocial behavior from the recipient child to the initiating child can occur more readily in sessions after the latter has demonstrated prosocial behavior than in control sessions.
(b) If positive emotions influence the establishment of direct reciprocity, affiliative behaviors from the recipient child to the initiating child can occur more readily in sessions after the latter has demonstrated prosocial behavior than in control sessions. If so, direct reciprocity is established through positive emotions of receiving prosocial behavior, and the specific indirect effect of positive emotions will be significant (Fig 1).

## Materials and methods

### Participants

In this study, observations were conducted at a private nursery school, in Osaka prefecture, Japan. We used two classes, A and B, as the participants of our study, comprising children aged 5 and 6 (those who were to reach age 6 during that year). Thirty-eight children were enrolled in Class A (19 boys, 19 girls; average age in months: 67.5 months; SD = 3.6), and 32 children were enrolled in Class B (16 boys, 16 girls; average age in months = 67.3 months, SD = 3.7). Of these, we selected children who would be targeted for observation, according to the frequency of evoking prosocial behaviors during the preliminary observation. As the preliminary observation required a long period, in this study, we made observations in Class A only. As a preliminary observation, the observer conducted an event sampling of prosocial behaviors for ten days inside Class A. Event sampling is a method of observing the target group as a whole, and each time a specific behavior occurs, a record is made of which individuals were related to the occurrence of the behavior [27]. As a result of the preliminary observations of the children who frequently demonstrated prosocial behaviors, we selected twelve (6 boys, 6 girls, mean age: 69.2 months; SD = 3.2) as children targeted for focal observation. Throughout the observation, all the children enrolled in Classes A and B were potential recipients of the focal children’s prosocial behaviors. The two classes had four full-time teachers engaged in childcare, and all the teachers were women. Before the observations, we explained the study’s objectives in writing to the parents and guardians of all the children in the two 5- and 6-year-old classes and obtained their written consent to carry out our observations. The research complied with protocols approved by the ethical committee in the fields of Psychology and Behavioral Sciences of the Graduate School of Human Sciences at Osaka University. This study adhered to the Code of Ethics and Conduct of the Japanese Psychological Association.

### Procedure

Observations were made over ten months, from June 20XX to March 20XX. An observer visited the nursery school four days a week and made observations between 9:30 a.m. and 4:30 p.m. Observations were made while more than half of the children enrolled in the class were playing freely, either inside the classroom or in the playground. No observations were made during activities outside the school, such as going for walks, and all observations were made between Monday and Friday.

During free play periods when observations were made, the children in Classes A and B played together, so they were free to come and go. The classroom had toys such as construction sets, building blocks, items for playing house, dolls, and picture books, and the children were able to play freely with them during the free play periods. To maintain consistency of the observational data, all observations were made by the first author, and a digital video camera (Sony; DCR-SR60) was used to make observations. The first author has built a favorable rapport with the children and nursery school teachers and carried out the main observations using the camera after the children became fully accustomed to being observed.

First, the observer used a 5-minute focal sampling method and observed the focal children in a random order. From the moment a focal child demonstrated prosocial behavior to another child, the observer made a focal observation of the child for ten minutes (Post-Prosocial; PP Setting). Prosocial behaviors were recorded if “object offering” or “helping” was evident. The definition of these prosocial behaviors was drawn up using Fujisawa et al. [5]’s definition for reference, and Table 1 illustrates the definition of each behavior. Of the types of prosocial behavior, those other than object offering and helping (e.g., comforting/consoling) have a low frequency of occurrence [5, 24], so they were not recorded in this study.

**Table 1:** Definitions of coded prosocial behaviors and affiliative behaviors.

Prosocial behaviors	
Object offering	Giving objects (e.g., a toy) to another child spontaneously, excluding cases in which the object is taken back within 1 minute.
Helping	Assisting another child to accomplish some goal spontaneously (e.g., putting on a smock, assisting a horizontal bar or pushing a bicycle)
Affiliative behaviors	
Hand-to-body	Touching another child’s body spontaneously, including the hand.
Body-to-body	Clinging to another child’s body spontaneously.
Talking	Talking to another child spontaneously, excluding verbal aggression (e.g., insults, derogatory comments). An instance of a child’s talking ended when the child stopped talking more than 5 seconds, and another instance began when the child started talking again.
Showing	Showing an object (e.g., a toy or a book) to another child spontaneously.
Approaching	Approaching within 1m of another child spontaneously, excluding cases in which the approach was accidental.

Moreover, for object offering and helping, we did not record them as prosocial behavior if the initiating child also benefitted (behaviors that had occurred as part of play); if the initiating child did it forcibly, against the recipient’s will; or if the recipient did not welcome such behaviors. The PP Setting’s 10-minute observation period was established, using, as a reference, a prior experimental study on direct reciprocity in early childhood [7]. We recorded the name of the focal child, the name of the child who was the recipient of prosocial behavior from the focal child, the name of the child who had been watching the prosocial behavior of the focal child within a 1-meter distance (if there were multiple children, all their names), and the location where observations had been made (inside the school or in the playground).

To compare with the PP sessions, we established a 10-minute control session (Matched Control; MC sessions). In this study, children who performed prosocial behaviors in the PP sessions were labeled as “initiating child” (always selected from among the focal children), and children who were the recipients of prosocial behaviors in the PP session were labeled as “recipient child”. The observer carried out observations of the MC sessions, with the following conditions:

(a) Observation of the MC sessions will be made between two and fourteen days after videotaping the PP sessions.
(b) Observation of the MC sessions will be made within two hours either before or after the start of PP sessions, to match the time zone with the PP sessions (if a PP session was videotaped, starting at 11 a.m., the MC session will be observed between 9 a.m. and 1 p.m. on a different day).
(c) An observation begins when a initiating child and a recipient child come close to each other (within 2 meters). This is to avoid the risk that if the initiating child is too far away from the recipient child, there may be few mutual interactions in the MC sessions.
(d) If a initiating child demonstrates prosocial behavior towards a recipient child during a 10-minute observation, the observation must be suspended to avoid the risk that the situation will become the same as in the PP sessions.
(e) Observations will be made in the same place as observations in the PP sessions (the location is matched: either inside the classroom or in the playground).

Regarding the abovementioned observation condition (a), this setting was to prevent the prosocial behavior seen in the PP sessions from influencing the child’s behavior in the MC sessions and preventing the relationship between an initiating child and a recipient child from changing drastically due to the interval between the PP and MC sessions becoming extended.

Regarding observation condition (c), the distance between an initiating child and a recipient child should have been unified to “within 1 meter” at the start of PP sessions. However, we prioritized the data collection efficiency and decided to include cases up to when the two children came to within 2 meters of each other. The observer recorded the distance between the initiating child and the recipient child at the start of MC sessions. In addition to analyzing all the sessions, the observer analyzed sessions, when an initiating child and a recipient child came closer, to between 1 meter of each other, in the MC sessions, and confirmed whether the setting of a distance used as a control had been reasonable. Regarding observation of the MC sessions, efforts were made to enhance validity as control data by rigorously controlling for the status of proximity between initiating children and recipient children, the location and time zone of the observations.

In both the PP and MC sessions, the observer carried out focal sampling and coded the prosocial behaviors from recipient child to initiating child (“object offering,” and “helping”), affiliative behaviors from recipient child to initiating child (“hand-to-body,” “body-to-body,” “talking,” “showing,” and “approaching”), and intervention by the schoolteacher with initiating children or to recipient children. Prosocial behaviors from recipient child to initiating child were also recorded, along with the time they had occurred. The definition of affiliative behavior was drawn up using that of Fujisawa et al. [5] for reference (“look,” which was regarded as an affiliative behavior in a study by Fujisawa et al., was eliminated, as it was difficult to record). Table 1 illustrates the definition of each behavior included in affiliative behaviors. As examples of interventions by nursery school teachers, we recorded behaviors such as those prompting recipient child to praise initiating child or to give a reward to initiating child.

The familiarity between initiating child and recipient child was assessed using an ethological method. Immediately after each PP and MC observation session was completed, the observer conducted a scan sampling and recorded the name of children within 1 meter of initiating child. For each initiating child, scan sampling was conducted an average of 509.33 times (range: 401–578). A proximity score was calculated for every possible combination in the following manner: (the proximity score of a pair = the number of sampling points at which the pair was observed in proximity /the total data points for the focal child). A study that targeted school-age children demonstrated that physical proximity correlated positively with familiarity [28]; subsequently, in this study, we used the proximity scores of each pair as an index of familiarity. Previous studies that employed similar naturalistic observations have confirmed the validity of using proximity scores as the index of familiarity [10, 11].

To assess the initiating child’s usual frequency of receiving prosocial behaviors, we measured the child’s focal sampling, independent of the PP or MC sessions. For each focal child, observations were made for 3,430 minutes. From these video data, we recorded the prosocial and affiliative behaviors that focal children had received from other children and calculated their frequency.

### Reliabilities

To confirm the reliability of data coded by the observer, approximately 10% of the video data targeted for analysis was randomly selected. A well trained research assistant other than the observer then coded them independently (10 min.×65 sessions). The following are Cohen’s Kappa values for each category: 0.80 for “object offering,” 0.82 for “helping,” 0.81 for “hand-to-body,” 0.79 for “body-to-body,” 0.78 for “talking,” 0.72 for “showing,” and 0.78 for “approaching.” Inter-observer reliability ranged from good to excellent.

### Analysis

We investigated whether differences were seen between the PP and MC sessions regarding prosocial and affiliative behaviors demonstrated by recipient child to initiating child. For analysis, we employed the Generalized Linear Mixed Model (GLMM; [29]), using the PP-MC pair as the Within-Subject Factor. In the analysis, we applied the same models 2 times using all datasets and the filtered data sets (Analysis 1, 2).

Analysis 1 used all datasets. Two models were investigated, using the data for 622 sessions (PP sessions: 311; MC sessions: 311). This data comprises 131 pairs of initiating child and recipient child, with 12 children being included as initiating child and 54 children as recipient child.

In Model 1, a model of prosocial behavior, “the presence or absence of prosocial behavior from recipient child to initiating child” was the dependent variable with a binomial error structure and logit link function. We then set “context” (PP vs. MC), “familiarity between initiating child and recipient child,” and “initiating child’s usual frequency of receiving prosocial behaviors” as the independent variables to study the fixed effects, and included “initiating child’s ID” and “recipient child’s ID” as the random effects. Including the IDs of initiating child and recipient child as random effects made it possible to manage the lack of data independence, such as recording multiple PP-MC pairs in the same dyads or different dyads formed by the same children.

In Model 2, a model of affiliative behavior, “the number of affiliative behaviors by recipient child to initiating child” was the dependent variable with a Poisson error structure and log link function. We then set “context” (PP vs. MC), “familiarity between initiating child and recipient child,” and “initiating child’s usual frequency of receiving affiliative behaviors” as the independent variables to study the fixed effects. The random effects were the same as Model 1.

Analysis 2 used the dataset filtered, to control three possibilities: the possibility of imitation, the influence of control distance at the starting point of the MC observations, and the effect of class. It is possible that recipient child was simply imitating another child’s prosocial behavior toward initiating child. The tendency to imitate peers’ prosocial behavior has been reported [21–23]. If recipient children took their prosocial or affiliative behaviors by imitation, it would not be evidence of direct reciprocity. Therefore, to consider the effects of imitation, we used the dataset that eliminated the sessions in which another child had demonstrated prosocial behavior to initiating child before recipient child had done so.

We also had to take into consideration of a difference in the control distance between initiating child and recipient child at the start of PP and MC observations. We needed to consider the possibility that the difference in the control distance between initiating child and recipient child at the start of the observations influenced the results. Analysis 2 was made using dataset comprising only those sessions in which initiating child and recipient child had come to within 1 meter of each other at the start of the MC sessions.

In the selection of the focal observation, whereas focal children were chosen from Class A, potential recipient children could be individuals who were in either Class A or Class B. Subsequently, there was concern that the degree of usual frequency of prosocial behaviors on the class level influenced the results, and the possibility that the frequency of mutual interactions and familiarity differed between the pair in which initiating child and recipient child were in the same class and the pair in which initiating child and recipient child were in a different class. Therefore, Analysis 2 was made using dataset comprising only the sessions that made observations of the group in which initiating child and recipient child belonged to the same class (Class A).

Controlling the above three possibilities, we conducted Analysis 2 using the dataset for 258 sessions (PP sessions: 129; MC sessions: 129) and investigated two models. This dataset comprised 57 pairs of initiating child and recipient child, with 12 children being included as initiating child and 26 children as recipient child.

Consequently, significant patterns and tendencies were similar between Analysis 1 and 2. For brevity purposes, we listed only the results for Analysis 1 (the analysis that used all the data). SPSS 20.0 statistical software was used for performing both analyses.

### Examination of the specific indirect effect of positive emotions in the relationship between context and the establishment of direct reciprocity

In the mediation analysis, it is a prerequisite that the independent variable, the mediator, and the dependent variable are related. The correlation between the independent variable (context: PP vs. MC) and the dependent variable (direct reciprocity: the presence or absence of prosocial behavior from recipient child to initiating child) was significant (*r* = .54, *p* < .001). Furthermore, the correlation between the independent variable and the mediator (positive emotion: the number of affiliative behaviors by recipient child to initiating child) was significant (*r* = .33, *p* < .001). The results clearly showed a relationship between the independent, mediator, and dependent variable, confirming the premise for performing the mediation analysis. 95% confidence interval (CI) was calculated using the bootstrap method to assess the specific indirect effect of positive emotion. The bootstrap method has been demonstrated and used by numerous studies in recent years [30]. HAD statistical software [31] was used for performing the analysis.

### Comparison of the temporal distribution of the first prosocial behavior of recipient child to initiating child between PP and MC sessions

If a prosocial behavior is seen from initiating child to recipient child, and the subsequent prosocial behavior from recipient child to initiating child occurs in relation, the distribution of time from the point when prosocial behavior is seen from initiating child to recipient child to the point when the first prosocial behavior is seen from recipient child to initiating child appears to differ from the distribution in the controlled sessions. We, therefore, compared the temporal distribution until the first prosocial behavior was seen from recipient child to initiating child, between all the PP and MC sessions in which prosocial behaviors were seen from recipient child to initiating child. The analysis was carried out using the Kolmogorov-Smirnov test, and R version 2.15.2 (R Core Team, 2012) statistical software was used for this analysis.

## Results

### Descriptive data

A total of 311 PP-MC pairs were observed (mean: 25.92 PP-MC pairs per focal child; range: 23–32 PP-MC pairs). In 622 sessions (311 PP-MC pairs), 12 initiating children and 54 recipient children were observed. 131 initiating-recipient dyads were observed. The familiarity between these dyads was estimated from the proximity scores (mean = 0.06, *SD* = 0.06). Prosocial and affiliative behaviors from recipient child to initiating child were observed more in PP sessions (prosocial behavior: mean = 0.78, *SD* = 1.05; affiliative behavior: mean = 5.18, *SD* = 3.99) than in MC sessions (prosocial behavior: mean = 0.04, *SD* = 0.22; affiliative behavior: mean = 2.60, *SD* = 3.44). From observational data independent of the PP or MC session, we assessed the initiating child’s usual frequency of receiving prosocial behavior (mean per session = 1.33, *SD* = 0.36) and usual frequency of receiving affiliative behavior (mean per session = 15.08, *SD* = 2.56).

As factors that influence the behavior of recipient child, we recorded the nursery school teacher’s interventions in either recipient child or initiating child. Throughout the observation, however, no interventions were seen by nursery school teachers in prosocial exchanges between the children. This appears to have been because these teachers did not often intervene in exchanges between 5- and 6-year-olds, except when conflict took place. We, therefore, judged that there was no need to consider the influence of interventions by nursery school teachers in the analyses.

### Analysis 1 (using all data): Model 1

**Fig 2:**
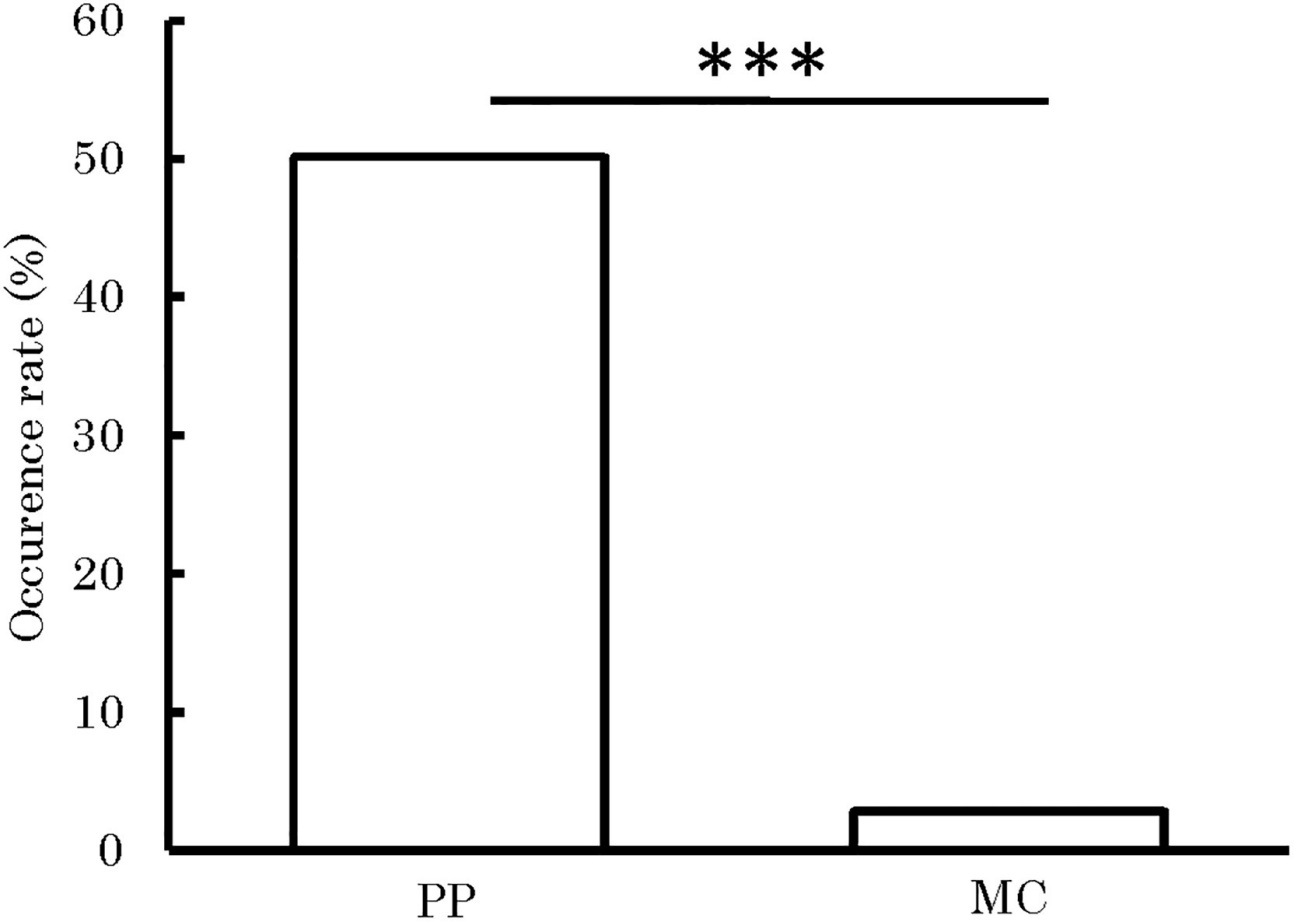
The occurrence rate of prosocial behavior from recipient child to initiating child in PP and MC sessions. Data are represented as session means of the occurrence rate of prosocial behavior for each context. ***: *p* < .001. The *p* value was calculated from the estimated value in Analysis 1 (Model 1).

As a result of analyzing Model 1, even if familiarity between initiating child and recipient child and initiating child’s usual frequency of receiving prosocial behaviors were simultaneously included as independent variables, the effects of the context (PP vs. MC) significantly influenced the presence or absence of prosocial behavior from recipient child to initiating child (Table 2). The recipient’s child’s prosocial behavior toward initiating child was seen with a significantly higher probability in the PP session (mean percentage = 50.16, *SD* = 50.08) than in the MC session (mean percentage = 2.89, *SD* = 16.79). Fig 2 illustrates the occurrence rate of prosocial behavior from recipient child to initiating child in both the PP and MC sessions.

**Table 2:** Influence of independent factors on the proportion of sessions in which prosocial behavior were occurred from recipient children in Analysis 1, in Model 1.

Independent term					
Factors	Level	Coef	SE (coef)	*t*	*P* (>|*t*|)
Intercept		−0.83	0.45	−1.83	0.07
Context	PP	3.56	0.35	10.18	<0.001
Familiarity between initiating children and recipient children		3.11	1.43	2.18	0.03
The initiating children’s usual frequency of receiving prosocial behavior		0.07	0.05	1.26	0.21

We analyzed the data in 622 sessions (311 PP-MC pairs, initiating child = 12, recipient child = 54, initiating-recipient dyad = 131) in Analysis 1, Model 1. The generalized linear mixed model with binomial error structures was used in the analysis. In the linear model with categorical independent variables, one of the levels was treated as a criterion, and the parameters of the other levels were estimated as the difference from the criterion level. In this model, in the factor "context", the level "MC" was treated as a criterion, and the coefficient of "PP" was shown as the differences from the level of "MC".

Familiarity between initiating child and recipient child also significantly influenced the presence or absence of prosocial behavior from recipient child to initiating child. This result demonstrates that the more familiar between initiating child and recipient child, the more likely recipient child was to demonstrate prosocial behavior to initiating child. Initiating children’s usual frequency of receiving prosocial behaviors did not influence the presence or absence of prosocial behavior from recipient child to initiating child.

### Analysis 1 (using all data): Model 2

**Fig 3:**
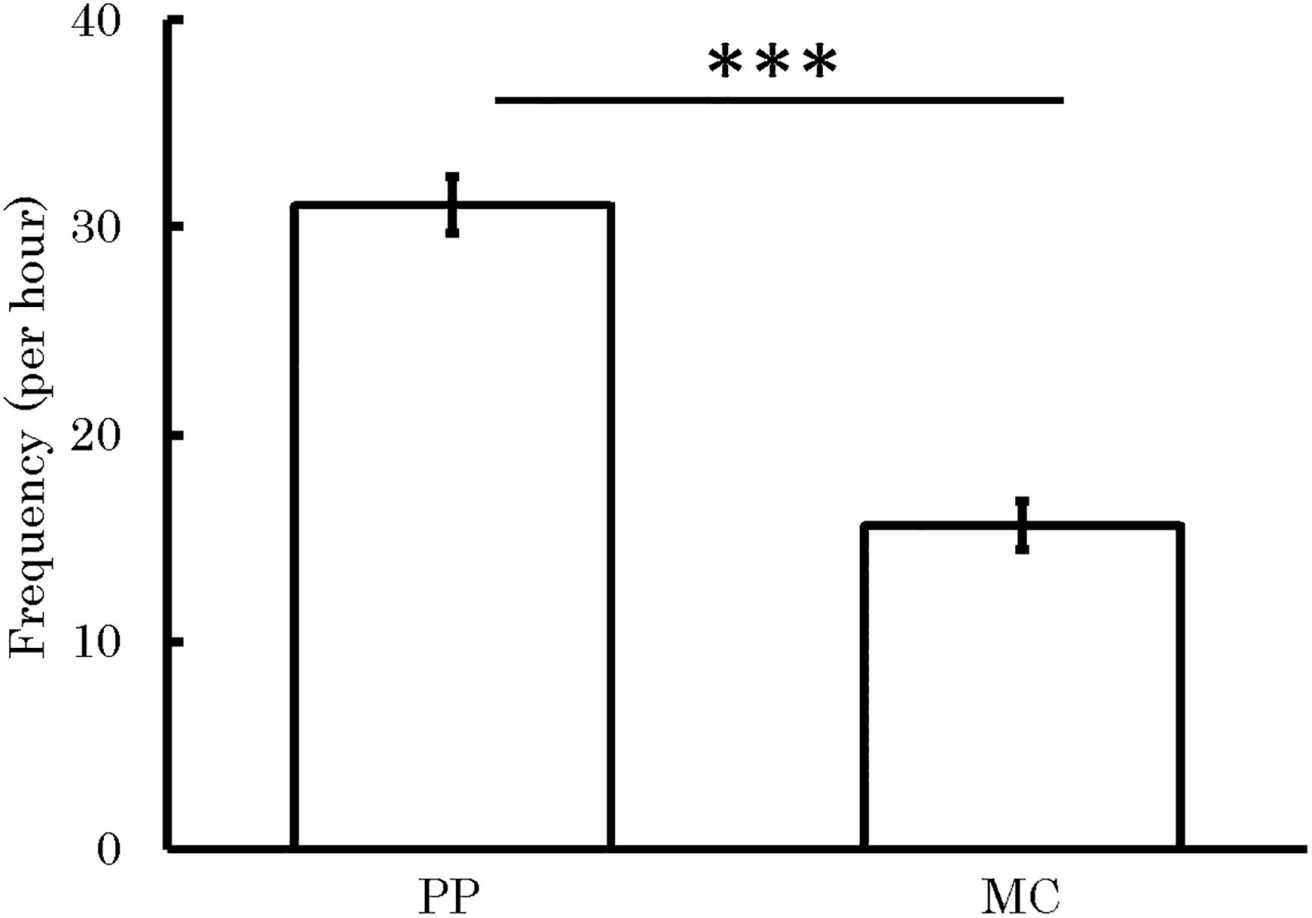
Frequency of affiliative behavior from recipient child to initiating child in PP and MC sessions. Data are represented as session means ± 1 SE of the frequency of affiliative behavior for each context. ***: *p* < .001. The *p* value was calculated from the estimated value in Analysis 1 (Model 2).

As a result of analyzing Model 2, even if familiarity between initiating child and recipient child and initiating child’s usual frequency of receiving affiliative behaviors were simultaneously included as independent variables, it was clear that the context (PP vs. MC) had significantly influenced the frequency of affiliative behaviors from recipient child to initiating child (Table 3). In other words, even if we controlled for the effects of familiarity between initiating child and recipient child, as well as initiating child’s usual frequency of receiving affiliative behaviors, the effects of the context had influenced the frequency of affiliative behaviors from recipient child to initiating child. The recipient child’s affiliative behaviors to initiating child were significantly higher in PP sessions (mean frequency per hour = 31.06, *SD* = 23.94) than in MC sessions (mean frequency per hour = 15.61, *SD* = 20.66). Fig 3 illustrates the frequency of occurrence of affiliative behaviors from recipient child to initiating child in both the PP and MC sessions.

**Table 3:** Influence of independent factors on the number of affiliative behavior were occurred from recipient children in Analysis 1, in Model 2.

Independent term					
Factors	Level	Coef	SE (coef)	*t*	*P* (>|*t*|)
Intercept		1.35	0.25	5.32	<0.001
Context	PP	0.69	0.08	8.99	<0.001
Familiarity between initiating children and recipient children		3.55	0.52	6.83	<0.001
The initiating children’s usual frequency of receiving affiliative behavior		−0.001	0.003	−0.43	0.67

We analyzed the data in 622 sessions (311 PP-MC pairs, initiating child = 12, recipient child = 54, initiating-recipient dyad = 131) in Analysis 1, Model 2. The generalized linear mixed model with Poisson error structures was used in the analysis. In the factor "context", the parameters were shown in the same way as Table 2.

The effect of familiarity between initiating child and recipient child also significantly influenced the frequency of affiliative behaviors from recipient child to initiating child. The more familiar between initiating child and recipient child, the more likely recipient child was to demonstrate affiliative behaviors to initiating child. Initiating child’s usual frequency of receiving affiliative behaviors did not influence the frequency of affiliative behaviors from recipient child to initiating child.

### The specific indirect effect of positive emotions

**Fig 4:**
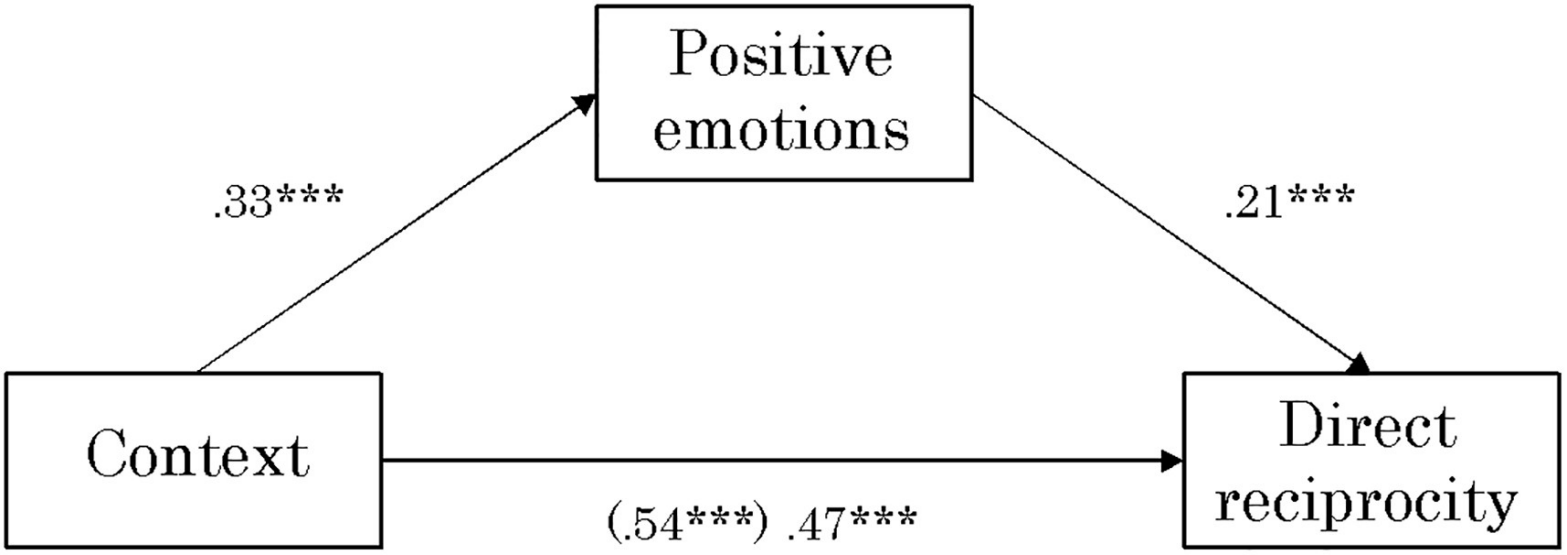
The effect of context on direct reciprocity through positive emotions (*n* = 622). ***: *p* < .001. The values in parentheses represent direct effect before controlling a mediator.

From Fig 4, the direct effect from context to direct reciprocity was significant (.54, *p* < .001), but when positive emotion was incorporated as a mediator, the direct effect decreased to .47 (*p* < .001). The value of the specific indirect effect is judged to be significant if the 95% confidence interval does not cross 0 and is not significant otherwise. The specific indirect effect was significant at .07 (95% CI: .04-.09), suggesting that positive emotion mediates the association between context and direct reciprocity.

### Comparison of the temporal distribution

**Fig 5:**
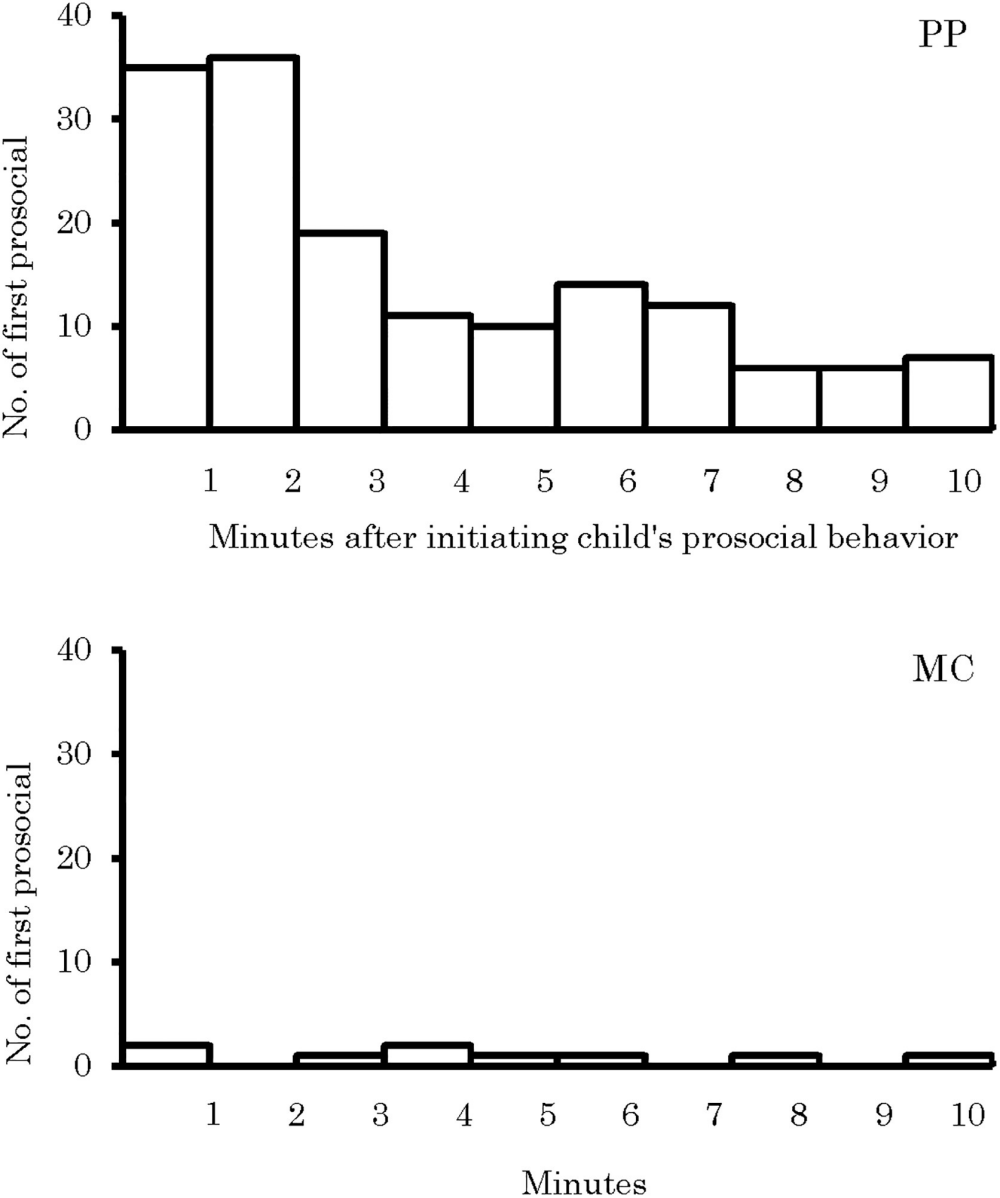
Number of recipient child’s first prosocial behavior to initiating child within 10 minutes of initiating child’s prosocial behavior.

As a result of comparing the temporal distribution of the first prosocial behavior of recipient child to initiating child between PP and MC sessions, we found a difference (Fig 5; Kolmogorov-Smirnov test, Dmax = 0.21, n1 = 156, n2 = 9, p < 0.001). The shape of the distribution in the PP sessions illustrated in Fig 5 is close to the negative exponential distribution, and, compared to the MC sessions, a difference was seen in the distribution up to the 7 minute after initiating child’s prosocial behavior toward recipient child was seen. It became clear that, compared to the control sessions, in sessions after having been the recipient of prosocial behavior from initiating child, recipient child returned the first prosocial behavior to initiating child more frequently during 7 minutes.

## Discussion

This study aimed to investigate whether 5- and 6-year-old preschool children establish direct reciprocity in the short term, using a naturalistic observation. Compared to the control sessions, the results demonstrated that young children were likely to demonstrate prosocial behaviors to other children after having received such behavior from them. Comparing the temporal distribution of returning prosocial behavior taken in both sessions, it demonstrates a difference in the distribution of up to seven minutes. In other words, 5- and 6-year-olds often return prosocial behaviors received from other children within the short period and are likely to quickly establish direct reciprocity. These results demonstrate that young children’s prosocial behaviors were being exchanged, in the short term, in a format that coincided with the mechanism of direct reciprocity. This is the first evidence to demonstrate direct reciprocal exchanges in response to peer’s behavior in natural interactions with their peers.

Throughout the observation, no interventions were seen by nursery school teachers in which they praised prosocial children or prompted nearby children who were observing to give any rewards to prosocial children. In other words, direct reciprocity seen between young children had been established spontaneously, without instruction from adults.

At the ages that were targeted in this study, the occurrence of prosocial behavior is likely to be influenced by the familiarity between children [7, 17] and the child’s personality [14, 32–34]. Due to this, in addition to the effects of the context that are the major consideration points (PP vs. MC), we simultaneously included, as independent variables in our analyses, two factors that appear to exert a significant influence on the occurrence of prosocial behaviors, namely, the familiarity between the initiating child and their partner in the exchange, as well as the initiating child’s usual frequency of receiving social behaviors. The results demonstrated that, even if the effects of familiarity and usual frequency of receiving social behaviors were controlled for, the effects of the context were significant.

In this study, the observation method, which set up control sessions, made it possible to make comparisons that used the PP-MC pairs as Within-Subject Factors. Moreover, by employing the GLMM as the analysis method, it became possible to consider the self-correlation between each PP-MC pair, allowing us to deal with the influence that individual differences between the focal child and their exchange partner had on the overall results. As seen, while using the naturalistic observation method, this study can be said to have identified the establishment of direct reciprocity in the exchange of prosocial behaviors by taking into consideration, as far as possible, the influence of individual differences such as the child’s personality as well as relationships between the children.

We believe that using naturalistic observations in a nursery school, where natural mutual interactions between children can be seen, gives the results of this study high ecological validity. However, studies that use naturalistic observations require a long time for collecting data, so it was not easy to gather sufficient data to withstand quantitative analyses. To increase data collection efficiency, a difference arose in controlling the distances between the children at the start of observation in the PP and MC sessions. We also selected children who were observation targets from among just one of the two classes. The possibility of differences in control distances and bias in the class from which focal children had been selected influencing the effects was investigated in Analyses 2. The results demonstrated that Analyses 2 did not differ from the analyses using all the data sets in terms of major tendencies. Therefore, it is believed that the differences in control distances and the bias of classes from which the focal children had been selected did not influence the results. Based on these results, as demonstrated in previous experimental studies [6–8], we conclude that preschoolers also establish direct reciprocity over the short term in their natural exchanges with their peers.

Previous experimental studies have pointed out that, if prompted by adults or within different pairs, young children begin demonstrating direct reciprocity corresponding to the other person’s behaviors from early childhood [6, 7]. According to a study targeting 3- to 7.5-year-old children that investigated, in a cross-sectional manner, the establishment of direct reciprocity in experimental settings, by the time they turn 5.5 years old, children can be seen to begin establishing direct reciprocity corresponding to the other child’s behaviors in a stable fashion, without being promoted by adults [8]. Our study reveals that, in the context of natural exchanges between 5- and 6-year-old children, direct reciprocity was established. An examination of these results, together with the results of House et al. [8], demonstrates that, when a child reaches 5 or 6 years of age, direct reciprocity is established, both in controlled experimental settings as well as in natural settings, whereby they quickly return prosocial behavior to another child once having been demonstrated such behavior by the latter.

It has been pointed out that, as the reason for prosocial behaviors, preschool children tend to make self-focused rationalizations, such as thinking whether the other child will return a favor in the future [35]. Additionally, according to a study that asked children who had spontaneously carried out a prosocial behavior at a nursery school about their motivation for the act, 4- to 5-year-olds reportedly rationalized their prosocial behaviors by linking them to direct reciprocity [25]. We also know that when a child reaches about 6 years old and tackles a task to share an object, they explain the reason for doing so in terms of the other child’s previous behaviors towards them [36]. If we were to use this knowledge and findings as the basis, there is a possibility that ages around 5 and 6 may represent a period when the children’s prosocial exchanges are likely to become reinforced by direct reciprocity. However, in our study, we used only 5- and 6-year-olds as our subjects and did not compare other age groups. Thus, we cannot conclude if the direct reciprocal exchanges seen in prosocial behaviors are indeed a tendency that begins to be seen from around ages 5 and 6. To identify the developmental changes in direct reciprocity between young children in natural settings, we need to investigate the content of prosocial exchanges between children while following their course of development.

Fujisawa et al. [5] carried out behavioral observations for one year in a group childcare setting and reported that the number of prosocial behaviors exchanged between pairs of 3- and 4-year-olds correlated if seen over the long term. In contrast, House et al. [8] conducted a cross-sectional study using 3- to 7.5-year-olds as their subjects and demonstrated that, until a child reaches around 5.5 years of age, direct reciprocity of prosocial behaviors that correspond to the other child’s previous behaviors is not established. A difference is seen between these studies in the period in which direct reciprocity becomes possible. Regarding this difference, House et al. [8] state that, in direct reciprocity that becomes established over the long term, it is not possible to declare how a young child has behaved towards each prosocial behavior received, suggesting the possibility that direct reciprocity that corresponds to the other person’s behavior is not yet established at around ages 3 and 4. The possibility can be considered that the direct reciprocity being reported in 3- to 4-year-olds is a “direct reciprocity based on attitude” that is established over the long term, while being influenced by stable relationships with their peers, such as through friendships. With the subsequent development of cognitive ability, from around ages 5 and 6, contingent direct reciprocity appears to be established based on the other person’s previous behaviors. In our study, compared with the control sessions, young children were likely to demonstrate affiliative behaviors, identical to prosocial behaviors to their exchange partners in sessions after having been the recipient of prosocial behaviors from their exchange partners. In light of the knowledge that young children are likely to hold positive emotions towards others who direct affiliative behaviors towards them [26], if they receive prosocial behaviors from their exchange partners, they appear to hold positive emotions towards them, and this psychological change modifies short-term direct reciprocity. Mediation analysis also showed that the specific indirect effect of positive emotion was significant, suggesting that positive emotion act as a psychological process that promotes direct reciprocal exchange. Additionally, because the recipients of prosocial behaviors in this study often returned such acts to the initiating children quickly (7 minutes), the possibility can be considered that it is still difficult for 5- and 6-year-olds to memorize the content of exchanges precisely and for extended periods. Based on these facts, it is possible that the 5- and 6-year-olds have contingent, direct reciprocal relationship in a short-term, while tracking the benefits from their peers. The shortness of this term may indicate the limit of cognitive ability of 5- and 6-year-olds to track the benefits from peers. Contingency may be influenced by positive emotions evoked by the benefit of the previous partner, as children are also more likely to show affiliative behavior after receiving prosocial behavior. By exchanging prosocial behavior with positive emotions, children may gradually deepen their understanding of fairness.

Lastly, we will discuss cultural differences related to direct reciprocity. Whereas independence is regarded as important in Western culture, intra-group harmony and cooperation tend to be valued in Oriental cultures [37, 38]. In this study, no prompting from adults was observed, but it is pointed out that adults are often said to become involved with children to promote the development of prosocial behavior in Japanese culture [39]. Likewise, in providing discipline, Japanese mothers are demonstrated to be more likely than their American counterparts to involve themselves with young children to encourage them to read the emotions of other people who are sad/distressed [40]. If we consider that prosocial behavior is strongly influenced by socialization [24, 34], attention must be paid to these cultural differences. Young Japanese children likely establish direct reciprocity in prosocial behaviors in formats different from young children in Western culture. Cultural comparison studies have pointed out that Japanese university students, compared to American and Thai students, tend to feel debt as well as positive emotions that lead to gratitude after receiving assistance [41, 42]. Even direct reciprocity in prosocial behavior, which we have examined in this study, may follow different developmental paths in Western and Oriental cultures, and the factors that influence direct reciprocity may also differ. By examining the cultural differences that can be seen in direct reciprocity, we can identify universal aspects in the development of direct reciprocity and aspects that are likely to be influenced by socialization and can offer knowledge that is important, when considering if direct reciprocity is universal, to human’s cooperative relations.
